# Method for thermal evaluation of automotive gearbox packages taking into account load point-dependent oil distribution

**DOI:** 10.1007/s41104-022-00109-5

**Published:** 2022-06-30

**Authors:** Roland Uerlich, Theo Koch, Heiner Theising, Lutz Eckstein

**Affiliations:** Institute for Automotive Engineering (Ika), RWTH Aachen University, Aachen, Germany

**Keywords:** Thermal network, Gearbox, Oil distribution, SPH, Thermal analysis

## Abstract

The spread of all-electric drives is steadily increasing in all sectors of road transport. Due to the constantly increasing demands on efficiency and performance by legislation and customers, it is necessary to continuously push the system limits of the powertrains. This paper presents an approach that performs an initial thermal system analysis based on a first gearbox configuration and efficiency calculation. Here, the componentwise loss calculation is used to identify thermal hotspots within the gearbox stage. The basis of this analysis is the thermal network method. For the approach, the gearbox elements gear, bearing, shaft, seal and housing are broken down into standard thermal elements to be created automatically for any subsequent gearbox configuration. The linking of these elements to each other is also standardised and automated comparably. An extensive simulation study is carried out using the smoothed particle hydrodynamics method to consider the load point-dependent oil distribution, which enables an initial estimate of the oil distribution. The thermal network filled in this way is then solved on a time-step basis, allowing dynamic load cases to be considered. The quality of the method is validated within the paper using the VW ID 3 gearbox as an example. Due to the use of a series gearbox, the validation was carried out on the basis of the accessible housing temperatures. These already show good convergence of the method compared to other existing approaches, which reinforces the necessity of conducting further experimental studies.

## Introduction

Since January 2020, the EU Regulation (EU) 2019/631 has been the legal basis for the assessment of CO2 fleet limits. These limits describe the emissions of a manufacturer's vehicle fleet and, therefore, do not have to be complied with for each individual vehicle [7]. To comply with these regulations and to counteract possible financial penalties, car manufacturers are increasingly using hybridised and fully electrified drive systems in their vehicle fleets. These efforts have resulted in electric vehicle sales currently increasing despite the overall decline in vehicle sales during the COVID 19 pandemic [19].

An essential criterion for increasing the popularity of battery electric vehicles is the increase of the usable range. Compared to conventionally powered vehicles, this vehicle characteristic is gaining significance due to the required charging time. In addition to the battery characteristics, the efficiency of the vehicle drivetrain is crucial for the range of these vehicles [25]. While conventional powertrains require multi-speed transmissions to achieve the required driving characteristics, all-electric powertrains can often deliver the required performance without a multi-speed transmission due to the torque characteristics of the selected electric machine. In these cases, the electric machines are not always operated at their optimum efficiency, however. A significant improvement in overall efficiency can already be achieved by integrating a single- or two-speed gearbox. The topic was also taken up in the studies by Sorniotti et al. and Ahssan et al. Both sources point out that a compromise must always be found between achievable efficiency gains, additional system complexity and costs. [2, 22]

In contrast to the powertrains of conventionally powered vehicles, manufacturers can rely on a much smaller pool of empirical information in the field of electrified powertrains. For this reason, methods are required to estimate the interactions and the resulting performance of the system as a whole. The more simplified system topologies of the one to two-stage gearboxes tend to allow a high degree of automation and integration of these design methods between the components of the drive train. The proximity of the gearbox to the electrical machine as a heat source and the increased heat generation of the gearbox due to the higher system speed lead to a more complex thermal situation for these single and multi-stage gearboxes. These thermal conditions are discussed in Gronwald and Kern and in Li et al. However, the thermal analysis of the adjacent gearbox is not addressed [9, 14]. A possible approach for the analysis of such a system is presented in this paper.

## Overview of thermal modelling approaches for automotive powertrain modules

Determining critical thermal conditions in gearboxes is crucial for preventing material failures [10, 15, 17]. The thermal network method is an option to achieve this with a compromise of calculation time and calculation accuracy. These function similar to electric networks with nodes and links. The temperature difference between nodes (analogue to voltage differences) causes heat flows to occur. The relation of temperature difference heat flow (analogue to electric current) is affected by thermal resistances [17]. Thermal elements or nodes are assumed to be isothermal. This approximation leads to inaccuracies using the thermal network method. Increasing the number of nodes for the modelled system decrease the calculation inaccuracies.

### Current approaches to thermal powertrain modelling using the thermal network method

State of the art for calculating gearbox temperatures is the standard ISO/TR 14179. Power losses of the components and heat dissipations through the housing surface, gearbox foundation and protruding shaft segments are calculated and compared. Within the norm, the temperature is iteratively determined at which the equilibrium state between power loss and dissipated heat is reached. Due to the nature of the calculation approach, this method allows only stationary system states to be considered. Since solely one oil temperature is calculated, this approach does not offer information on critical thermal conditions for specific locations in the gearbox.

An extensive analysis of the heat balance of a gearbox using a thermal network was introduced by Geiger. A test gearbox consisting of two shafts with two bearings and a helical gear on each shaft was examined. The shafts were divided into multiple segments, each representing one node; a gear was divided into three nodes (flank, tooth, gear body). Every other component, including the oil and the rectangular housing, was represented by one node each. In advance, the total power losses were calculated and linked to the respective nodes, which causes heat to be generated in the model. The total power losses in a gearbox (Eq. 1) consists of shares of the gears (*G*), bearings (*B*), sealings (*S*) and other sources of power losses (*X*) such as couplings or splashing of planet carriers. For gear and bearing losses there are load-independent (0) as well as load dependent (*P*) losses. In-depth calculations of these losses are presented by Niemann et al. [20]. The generated heat is mainly dissipated through the housing surface to the environment. In this model, the heat capacities of the thermal elements were calculated, which allowed a transient temperature calculation. The simulated temperatures were validated with measured data of the test gearbox. The difference between the simulated and the measured oil sump temperature was less than 5% up to 120 °C, proving a more accurate temperature development assumption than the calculation based on ISO/TR 14179-2 [8]:

1 $$ P_{L} = P_{L,G,P} + P_{L,G,0} + P_{L,B,P} + P_{L,B,0} + P_{L,S} + P_{L,X} $$

Takabi et al. took a closer look at analyzing roller bearings in a thermal network [23]. The bearing was divided into three nodes (inner ring, rollers and outer ring). The test setup consisted of an electric machine-driven shaft with a bearing and a tight housing lubricated by an oil sump. Calculations were shown to determine the thermal resistances between the nodes. The difference between the calculated and the measured oil temperature for the test setup was less than 4 K up to 60 °C, which also means good accordance.

In contrast to the component-oriented approaches presented above, a manual six-speed gearbox was analyzed by Changenet et al. using a thermal network with 44 nodes. The main focus was the prediction of accurate power losses by taking temperature-dependent oil viscosity into account, which is calculated with the node temperatures of the thermal network. This method shows benefits, especially for low oil temperatures compared to isothermal approaches, because differing local rises in temperature are considered [3].

### Approaches to analysing the oil distribution within a gearbox

In addition to oil viscosities, oil distribution within the gearbox also plays an essential role in the heat balance. Several approaches exist for modelling fluid distributions and associated losses in gearboxes. An overview is provided by Concli et al. The methods presented are divided into experimental and simulative approaches. Models based on empirical investigations show insufficient accuracy but can be established comparatively quickly. They are, therefore, primarily suitable for initial estimations.

The optimisation of numerical methods and the associated hardware have established them as state of the art in current development processes. Among the different numerical approaches, the author considers the smoothed particle hydrodynamics (SPH) method suitable for determining the primary lubricant flows. Due to the Langrangian approach, the SPH method allows the consideration of complex flow fields in terms of macroscopic flow effects with reduced computational effort compared. In contrast, the finite volume method (FVM) and computational fluid dynamics (CFD) combined with a suitable remeshing technique provide more accurate results in power loss prediction [4].

General potentials of the SPH method towards industrial applications are presented by Shadloo et al. The author clarifies the motivation of application, current state, and challenges of the continuously developing FVM and contrasts this with the SPH approach. In addition to a comprehensive discussion of the advantages and disadvantages of the two approaches, the author points out that the significant computing time advantages of the SPH method make it suitable for use in an industrial context. This description results in brief analysis possibilities for various flow problems, estimated with reduced modelling effort [21].

Emmer provided one of the first approaches using the SPH method to simulate oil flow inside gearboxes. In particular, different scenarios of spur gear arrangements and variations of boundary conditions are examined. The results are analyzed together with a CFD simulation and provide a comparable description of the general flow condition inside the gearbox. Based on the presented computational time advantage, the suitability of this approach for modelling free surface flows is manifested and demonstrated using the example of the complex flow field of an automotive gearbox [5].

A further extension of the approach is presented in Liu et al. An SPH-based simulation model is set up to investigate the oil flow and the churning losses of a dip-lubricated spur gear pair in a test gearbox. The results are discussed for various rotational speeds, lubricant viscosities and oil temperatures. It is shown that the SPH method provides a high potential for predicting the oil distribution in dip-lubricated gearbox systems. In loss prediction, discrepancies are evident, which indicates that the current approach is insufficient [16].

Similar investigations of SPH simulations inside gearboxes are presented by Ji et al. The computational results are compared to experimental particle image velocimetry (PIV). To investigate the flow field behaviour, different oil levels and Reynolds numbers are considered. The main examination points are the aeration effect due to the rotation of gears, the velocity field, its profile and the expression of the oil surface. In general, the simulation results demonstrate physically consistent behaviour of the oil flow and good illustration of flow structures, splashing and recirculation areas compared to PIV [11].

## Methodological approach to thermal modelling

The approach presented in this paper is based on an automatically generated thermal network. As stated before, thermal networks function similar to electric networks with nodes connected through links, defined by thermal resistances. Analogue to Ohm's law, the relation between the heat flow $\dot{Q}$, thermal resistance $R_{{{\text{therm}}}}$ and the temperature difference ${\Delta }T$ as the driving potential is given by Eq. (2). For further calculation, the conductivity value $L$ is introduced, which is reciprocal to the thermal resistance. The variables $i$ and $j$ describe the two nodes between which the thermal relationship is established:

2 $$ \dot{Q}_{i,j} = \frac{{T_{i} - T_{j} }}{{R_{{{\text{therm}},i,j}} }} = L_{i,j} \cdot {\Delta }T_{i,j} . $$

The type of heat flow determines the conductivity value. These are differentiated into conduction, convection or radiation. Within a gearbox, all components are in exchange via the internal radiation heat flows. The modelling of this effect would significantly increase the complexity and calculation time of the approach. As the effect on the global heat balance is considered to be rather small due to the compensation of the opposing heat radiation of the individual gearbox components within the housing, this is not considered and instead only the radiation of the housing with the environment is modelled. This simplification was already introduced in the work of Geiger. Due to the complexity of the calculation methods of the conductivity values for conduction and convection, these are not shown here in detail. The calculations follow the formulas and methods given in the VDI Heat Atlas, by Geiger and by Takabi et al. Table 1 lists typical values for thermal conductivities and heat transfer coefficients, on which the calculation of conductivity values for conduction and convection, respectively, is mainly based on [1, 8, 23].

**Table 1:** Typical values for thermal conductivities and heat transfer coefficients

Thermal conductivity, *λ* [W/km]		Reference heat transfer coefficient, *α* [W/km^2^]		Heat transfer coefficient, *α* [W/km^2^]	
0.015–0.15	Gasses	2–25	Free convection in gasses	10–25	Free convection between housing and ambient
0.1–0.65	Liquids	10–1000	Free convection in liquids	25–250	Forced convection between housing and ambient
1–450	Solids	25–250	Forced convection in gasses	50–5000	Convection between gears and oil
		50–20,000	Forced convection in liquids	50–2000	Convection between bearings and oil
		2500–100,000	Condensing or boiling liquids	50–1000	Convection between oil and housing

To solve the thermal network, the principle of Kirchhoff's law is applied equivalent to an electrical grid. Its adaptation to the characteristics of the thermal network can be seen in Eq. (3). In its application, the sum of all outgoing and incoming heat flows at a node equals the power loss occurring at the node:

3 $$ \mathop \sum \limits_{j,j \ne i}^{n} \dot{Q}_{i,j} = P_{i} . $$

Since this equilibrium only holds for steady-state conditions, the imbalance of the power balance of a node in the transient state causes the temperature of the node to increase in proportion to the heat capacity. The above assumes that there is no phase change of the gear components. Therefore, for the presented problems considered in this paper, a time-discrete solution formulated in Eq. (4) is established for the nodes of the thermal network. Hence, for a network with *n* nodes, *n *− 1 power balances are set up to form a solvable system of equations. The remaining node, for which no system of equations can be set up, usually represents the environment. The thermal mass of this node is large enough to assume a constant value. Thus, this node is considered a reference, and the temperature differences to the other nodes are calculated by solving the system of equations:

4 $$ P_{i} - \mathop \sum \limits_{j,j \ne i}^{n} \dot{Q}_{i,j} = C_{i} \frac{{T_{i,\tau + 1} - T_{i,\tau } }}{{t_{\tau + 1} - t_{\tau } }} = C_{i} \frac{{\Delta T_{i,\tau } }}{{\Delta t_{\tau } }}. $$

**Fig. 1:**
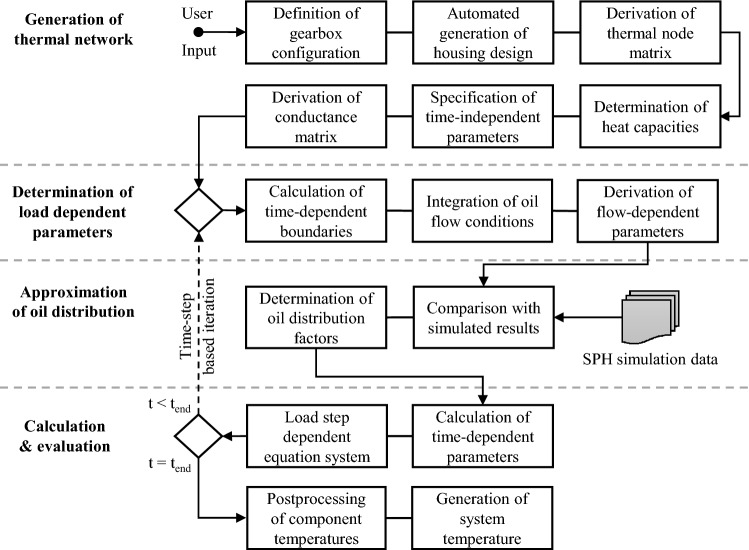
Flow chart of the thermal network method for analysing the gearbox temperature

In this paper a method is developed to set up the described thermal network for a gearbox, illustrated in Fig. 1. The methodology presented follows the approach to automated powertrain development already presented by Kieninger et al. and is an extension of the method developed at the Institute for Automotive Engineering (ika) regarding the conceptual design and evaluation of powertrain systems [13].

The method of thermal modelling is divided into four steps. These are: the generation of the thermal network, the determination of the load-dependent parameters, the approximation of oil distribution and the calculation of the system of equations. In the first step, the thermal network is set up. In this step, the gearbox configuration is initially converted into the notation required for further considerations. Based on this notation, a housing design can be created using the positions of the individual gearbox elements. The first draft of the thermal network, which considers the thermal connection between the individual components, is then derived from these specifications. This network is supplemented by the time-dependent and independent thermal variables so that a consistent conductance matrix can be generated. In the following process step, the load point-dependent simulation parameters are determined. In addition to the typical estimation of speed-dependent heat transfer coefficients, this includes quantifying those coefficients directly dependent on the oil distribution within the gearbox. These are subsequently determined using an existing data set derived from oil distribution simulations. This data set was developed using the SPH simulation approach. The approach is detailed in the following sections. In the last step, the time-dependent parameters are calculated for the respective time interval, which are mostly the conductivity values for convection and the needed heat transfer coefficients. Afterwards, the time-step-based solution of the thermal equation system is carried out. At this point, results of an advanced efficiency calculation of the system are used for the component power losses to be considered. The approach used has already been presented by Kieninger et al. and Uerlich et al. [12, 24].

### Basic model structure for the analysis of gearboxes

**Fig. 2:**
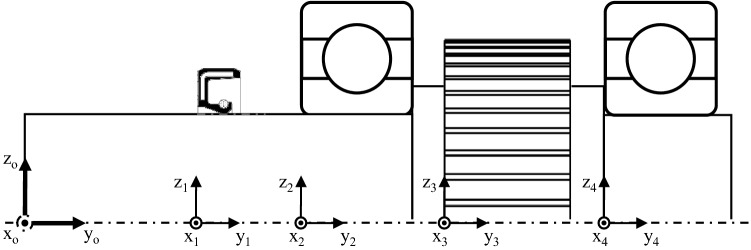
Representation of the global coordinate system of the shaft as well as the coordinate systems of the further gearbox elements of this shaft

Within the previously mentioned framework all components are arranged in a global coordinate system. In this coordinate system, for the typical application case of purely coaxial gear shaft arrangements, the *y*-axis is always placed in the direction of the shaft and the *x*- and *z*-axes point in the radial directions (horizontal and vertical direction, respectively), see Fig. 2. An origin point gives the position of an object in a three-dimensional space. The *x*- and *z*-coordinates of that origin are the centre of the object in the *x*–*z*-plane, and the *y*-coordinate is the lowest *y*-coordinate of the *y*-area the object covers. In Fig. 2, the origin of the coordinate system equates to the origin of the shaft. This central coordinate system is used to align the coordinate systems of all other gearbox elements of this shaft.

**Fig. 3:**
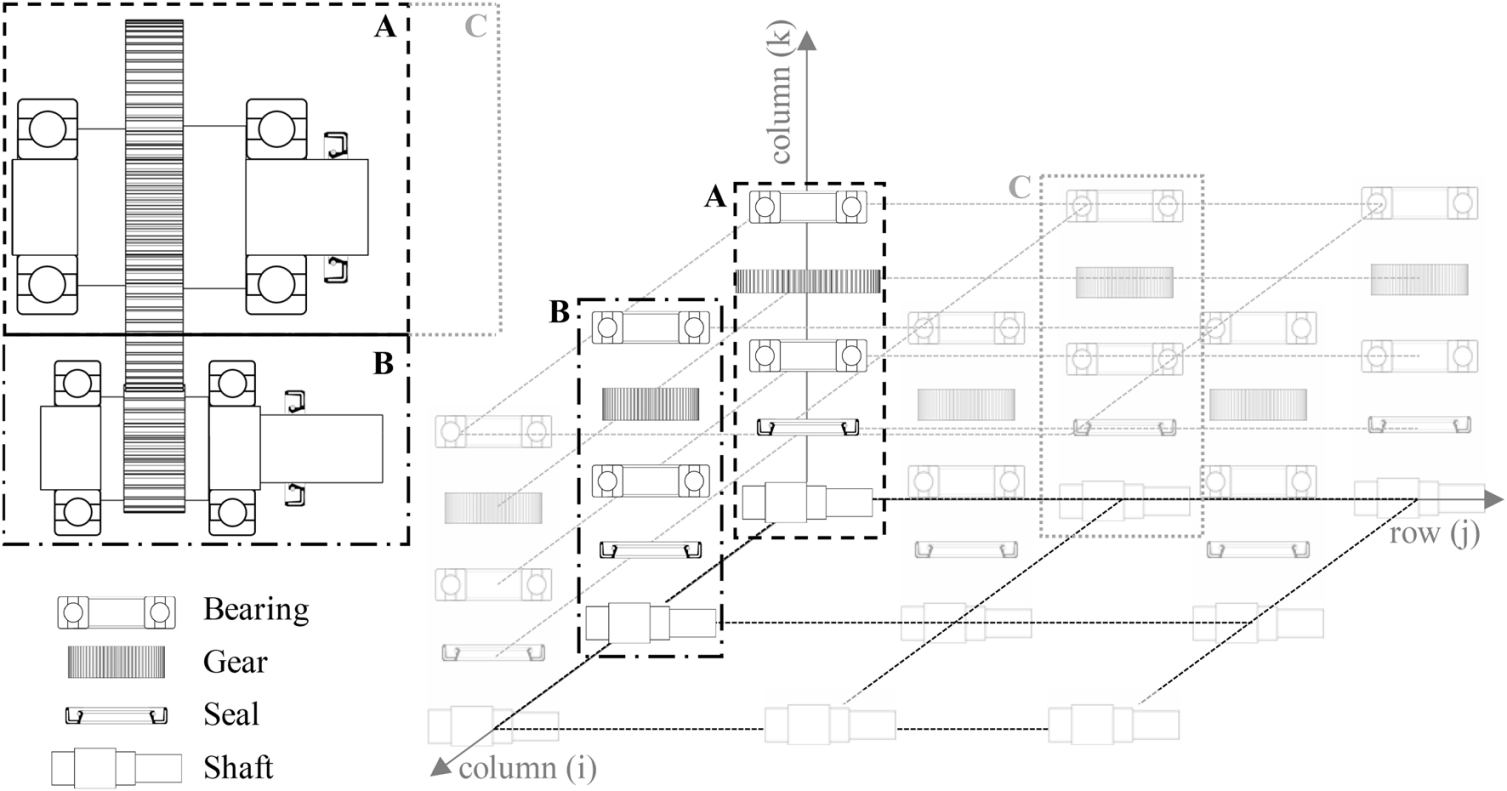
Generalised grouping structure for automated gearbox analysis

An example gearbox configuration is used for further explanations, shown in the top left corner in Fig. 3. The gearbox contains one helical gear pair with the input shaft in area B and the output shaft in area A. On each shaft, there are two bearings, one seal and one gear.

All components of the gearbox are subdivided into groups. The definition of the term groups is elaborated in the following. In general, there is one group for each shaft-system in the gearbox containing its components. If there are coaxial shafts, the shaft-systems coaxial to each other are combined and the group contains all components being assembled on these shafts. For instance, the example configuration is divided into two groups (in Fig. 3 marked as group A and group B). The components are organized in a three-dimensional matrix, which is exemplarily shown in Fig. 3. The gearbox components are stored as columns in this matrix. The second group B is axially parallel to the group A and, therefore, next to it along the *i*-axes. If there would be another shaft-system C (see Fig. 3), which is coaxial to the shaft-system A, the first group would contain both shaft-systems and the shaft-system C would be next to the shaft-system A along the *j*-axes. Therefore, the i-dimension indicates different groups, the *j*-direction different coaxial shaft-systems in the respective group, and the *k*-direction indicates different components and the shaft itself. Shafts are always listed in the *i*–*j*-plane, where $k = 1$. The shafts in a group and the components on a shaft are ordered by the ascending *y*-coordinate of the origin. Since there are no coaxial shafts in either of the two groups in the example gearbox configuration, there is only one column in the three-dimensional matrix for this particular configuration. The transparently displayed elements in Fig. 3 show what the matrix would look like for a possible more complex gearbox. Since neighbouring elements are easily apparent in the matrix, the structure allows a basic understanding of the geometric arrangement of the gearbox in the further analysis and simpler and shorter access of data of desired components. Thus, the structure is fundamental for the housing generation and the thermal analysis which is described in this paper, in particular for the creation of the inner surface of the housing and the automatic linking of the thermal nodes.

For each gearbox element except the oil and the air inside the gearbox, a three-dimensional volume element is automatically generated. The generation is conducted in two major steps. The first step is creating point clouds for each gearbox element based on generation scripts depending on the element type. In the second step, the point clouds are connected by a MATLAB function called alphaShape [18]. The volume models created this way are used to check if gearbox elements are colliding with each other. Likewise, they are used for surface and volume calculation. These parameters are then determined using MATLAB functions.

### Automated generation of simplified housings for thermal assessment

**Fig. 4:**
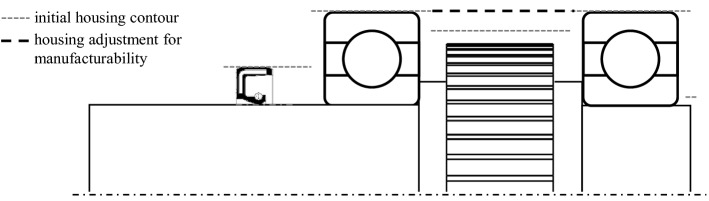
Determination of the inner contour of the group housing based on the components

To take into account not only the internal gearbox elements but also the housing and the air and oil enclosed in it, it is necessary to create a simplified gearbox housing. For this purpose, each element group of the gearbox is first considered individually. Again, two design steps are carried out. Here, the contour of all components of the group is considered. The approach can be seen in Fig. 4. The grey dashed lines mark the outline, in which the radial clearance for each component in the group is considered. This is equivalent to the tightest inner contour of the group housing. Since production, assembly and functionality of such a housing contour lead to difficulties, the inner contour is simplified to allow a reliable volume estimate. This simplification is represented by the black dashed line. The clean-up contour's key characteristic is that going from the section with the maximum radius sectionwise to the left and right, the radii are only becoming smaller. In this process, housing details such as bearing faces and oil collection patches are neglected to generate housings for a larger number of gearbox variants without carrying out a time-consuming modelling process. The outer contour is derived from the inner contour considering the housing thickness.

**Fig. 5:**
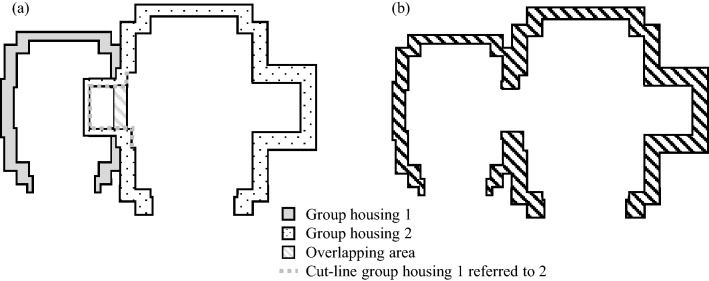
Merging the two group housings of the example gearbox

In the first step, each group housing is created and merged into one overall housing in the second step. If the point clouds of all group housings were merged, points of group housings would appear within the inner space. To avoid this, the point clouds are merged under various conditions. The general approach to redesigning the housing is shown in Fig. 5. The grey filled area represents the group housing for group 1 and the black dotted area is of group 2. A cut line is also marked with a grey dotted line. All points of the point cloud of group housing 1 located to the right of this line are deleted. The affected area is marked with grey hatching. This cut line is derived based on the inner contour of the group housing 2.

After this approach has been carried out for the affected points of group housing 1, it is repeated for the cloud of points of group housing 2. Here, the cut line is derived based on the inner contour of the group housing 1. If these adjustments result in gaps in the housing, these are closed in a further step, whereby the restrictions of each gearbox element remain in force. The point cloud created in this way serves as an initial estimate of the gearbox housing. Still, the estimates show considerable deviations from a later detailed structural design due to the nature of the forecast.

A final mesh is created again using the alphaShape function [18] based on the point cloud. The mesh is later used to derive the thermal properties of the housing and thus can estimate the thermal behaviour based on an initial gearbox configuration.

### Generation of thermal network

**Fig. 6:**
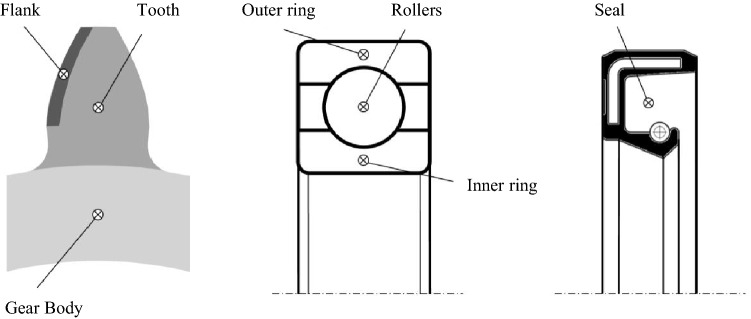
Thermal elements of gear, bearing and seal

To create the thermal network, standardised node sets are defined for each gearbox element and the thermal network is built up from these standard elements based on the gearbox configuration. Figure 6 shows the nodes in the thermal network for the considered gearbox components. A gear has three thermal elements: flank, tooth, gear body, as depicted in Fig. 6. A bearing is divided into the inner ring, the rollers and the outer ring. Seals are represented by one thermal node.

**Fig. 7:**
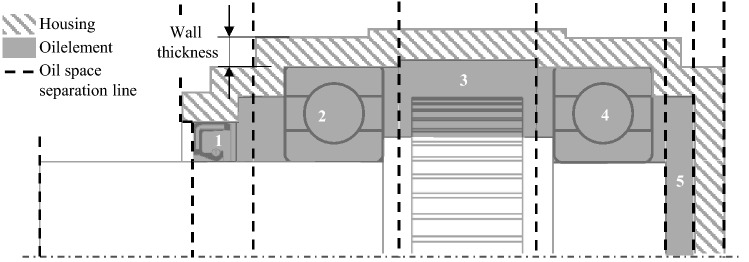
Illustration of the partition planes required for the oil space generation

In addition to the gearbox elements already mentioned, the gearbox oil–air mixture inside the gearbox is also considered within the thermal network. To create the necessary thermal nodes, the gearbox internal space is divided into subspaces. In this process, every group is considered separately. The general procedure of forming the subspaces is illustrated by the first group of the example gearbox in Fig. 7. In the first step, component zones are set up, determined by the gearbox elements' positions and predefined spacing depending on the element type. The internal space is cut between those component zones, which leaves boundaries. These boundaries define the subspaces of the oil–air mixture in the y-direction for the respective group. In the radial direction, they are bound by the housing or by the counter-oil-subspace of a spur gearpair. For gearbox configurations with planetary gearsets, additional oil spaces in the radial direction within a hollow cylinder shape are required, which will not be described here, since further explanations are based on a simple spur gear stage example. The oil subspaces and their properties are stored in a three-dimensional matrix similar to the group structure in Fig. 7. The first dimension in this matrix indicates the group. The second dimension indicates the position of the subspace in the group along the *y*-axis. The third dimension indicates the position of the subspace in the radial direction for planetary gear sets. Based on this data, it is then possible to specify the network’s thermal nodes of the oil–air mixture with the necessary characteristic values.

**Fig. 8:**
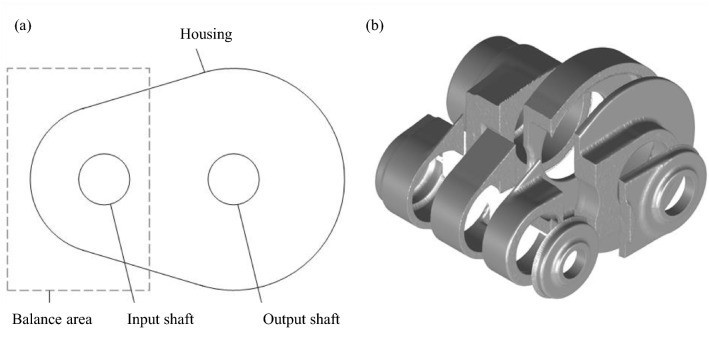
Schematic representation of the division plane (**a**) and a three-dimensional realization of the sectional elements (**b**)

In addition to the interior of the gearbox, its housing is also subdivided into several thermal nodes. The developed process is structured into two main steps. This process considers the groups separately to create the thermal elements for the oil–air mixture. To achieve this, the first step is cutting out a piece of housing for every group, leaving the combined pieces with no overlapping volumes. One fundamental part of that process is shown in Fig. 8. For shaping, the piece of the housing 1 group is looked at and compared to every other group. To be able to compare the groups, in this example a balance volume is placed in the centre of the *x*–*z* plane of group 1 and aligned to the *x*–*z* centre of group 2. This balance area covers the same area on the y-axis as the housing for this group. The distance of the balance volume is determined by considering the ratio of the maximum radii of the group housings, so that balance volumes of radially smaller housings are smaller and vice versa. When this is done for every possible pair of groups, an individual housing piece is provided for each group.

The aim of the second major step is the same as for creating the thermal elements of the oil–air-space. The same boundaries are used to cut the housing piece inside a group. This step finalises the creation of thermal elements of the housing. From these housing elements, the contact surfaces to the neighbouring elements and the mass and surface of the respective housing elements can be derived. The data generated for the respective housing elements are stored in a matrix based on the group structure to be accessed in a simplified manner in the later course of the thermal modelling.

**Fig. 9:**
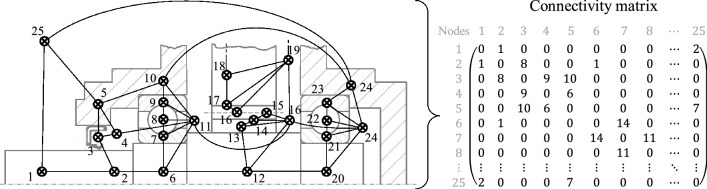
Representation of the thermal network based on a schematic drawing of the gearbox as well as the derived connectivity matrix

After all gear elements have been divided into thermal nodes by the process steps described, these nodes are interconnected. A connection of two nodes each is defined based on their thermal relationship. Whether two nodes have a thermal relationship is recognised according to their geometric contact area, which is automatically detected. Figure 9 illustrates this automatic connection process using the first group of the example gearbox, whereby the connection to the elements of the second group is only indicated. A connection matrix is created from this analysis, which contains the thermal connection of the different nodes to each other. In addition to whether a node is connected to another node or not, this matrix also contains direct information on the type of thermal connection between these nodes.

To identify the nodes, each node is assigned an individual identification number (node ID). Based on this node ID, the assignment of the connection matrix is definite. Overall, the presented approach currently considers the thermal connection types shown in Table 2. Since a connection type is always specified for each node, the connection matrix is always a symmetric $n \times n$ matrix, where $n$ is the maximum node ID equal to the total number of nodes in the network. In the following, the index $T_{C}$ is used for this matrix, as shown in Fig. 9. For instance, the first node in Fig. 9 has the index $\epsilon_{1}$ being an outer ring of a bearing and $\epsilon_{2}$ being a contacting housing segment, the entry $T_{{C,\epsilon_{1} ,\epsilon_{2} }}$ would be 16, since this is the ID for the connection type according to Table 2.

**Table 2:** Connection types and their identification numbers

Connection type	ID	Connection type	ID
Shaft segment—shaft segment	1	Roller—oil space	13
Shaft segment—ambient	2	Inner ring—shaft segment	14
Oil space—oil space	3	Inner ring—oil space	15
Housing segment—housing segment	4	Outer ring—housing segment	16
Shaft segment—oil space	5	Outer ring—oil space	17
Oil space—housing segment	6	Flank—oil space	18
Housing segment—ambient	7	Flank—sprocket	19
Seal—shaft segment	8	Sprocket—oil space	20
Seal—oil space	9	Sprocket—gear body	21
Seal—housing segment	10	Gear body—oil space	22
Roller—inner ring	11	Gear body—shaft segment	23
Roller—outer ring	12		

After the thermal network is conditioned, its solution is determined time step-based. Within these intervals, all energy flows between nodes are assumed as constant. Smaller intervals ${\Delta }t$ lead to higher accuracies of the calculation. Following Geiger, the principle of the thermal network for a gear stage was implemented according to Eqs. (5)–(12) [8]. According to this approach, a transient energy balance is established for each node $\epsilon$, which is used to determine the node temperature $T_{\epsilon ,t + 1}$ in the next time step $t + 1$:

5 $$ T_{ \epsilon ,t + 1} = T_{ \epsilon ,t} \cdot {\text{e}}^{{ - \frac{{L_{ \epsilon ,n,t} }}{{C_{ \epsilon ,t} }} \cdot {\Delta t}}} + \frac{{E_{ \epsilon ,t} }}{{L_{ \epsilon ,n,t} }} \cdot \left( {1 - {\text{e}}^{{ - \frac{{L_{ \epsilon ,n,t} }}{{C_{ \epsilon ,t} }} \cdot {\Delta t}}} } \right). $$

The total number of nodes, including the ambient, is $n$. Because the $n$th node is always the ambient, $L_{ \epsilon ,n,t}$ is the conductivity value for the connection between the node $\epsilon$ and the ambient for the time step $t$. In Eq. (5), the energy term $E_{ \epsilon ,t}$ is given by

6 $$ E_{ \epsilon ,t} = P_{V, \epsilon ,t} + \dot{H}_{{ \epsilon , {\text{CU}},t}} + \mathop \sum \limits_{{ \epsilon_{1} }} \dot{Q}_{{ \epsilon , \epsilon_{1} ,t}} + \mathop \sum \limits_{{ \epsilon_{2} }} \dot{H}_{{ \epsilon , \epsilon_{2} ,t}} . $$

This means that the remaining energy flow of power losses, enthalpy and heat flows results in the node warming up or cooling down. The sum of heat flows $\mathop \sum \limits_{{ \epsilon_{1} }} \dot{Q}_{{ \epsilon , \epsilon_{1} ,t}}$ for the time step $t$ is calculated by

7 $$ \mathop \sum \limits_{{ \epsilon_{1} }} \dot{Q}_{{ \epsilon , \epsilon_{1} ,t}} = \mathop \sum \limits_{{ \epsilon_{1} }} L_{{ \epsilon , \epsilon_{1} ,t}} \cdot \left( {T_{{ \epsilon_{1} ,t}} - T_{ \epsilon ,t} } \right). $$

The sum of enthalpy flows $\mathop \sum \limits_{{ \epsilon_{2} }} \dot{H}_{{ \epsilon , \epsilon_{2} ,t}}$ caused by flowing oil–air-mixture for the time step $t$ follows:

8 $$ \mathop \sum \limits_{{ \epsilon_{2} }} \dot{H}_{{ \epsilon , \epsilon_{2} ,t}} = \mathop \sum \limits_{{ \epsilon_{2} }} \dot{C}_{{ \epsilon , \epsilon_{2} ,t}} \cdot \left( {T_{{ \epsilon_{2} ,t}} - T_{ \epsilon ,t} } \right). $$

Using these formulas, the temperature for one node is calculated. To calculate the temperature for all nodes in the thermal network compactly, a system of equations is set up for every time step $t$, Eq. (9). The vector $\vartheta$ represents the temperature difference of each node compared to the reference node (ambient). $A_{{{\text{TA}}}}$ is the system matrix, and its entries describe all information about the heat exchange between nodes. In vector $b_{{{\text{TA}}}}$ the power losses of each node are considered:

9 $$ A_{{{\text{TA}},t}} \cdot \vartheta_{t} = b_{{{\text{TA}},t}} . $$

The matrix $A_{{{\text{TA}}}}$ has a size of $n - 1 \times n - 1$. Both $\vartheta$ and $b_{{{\text{TA}}}}$ is a $\left( {n - 1} \right) \times 1$-vector. The diagonal entry $A_{{{\text{TA}},t, \epsilon_{1} , \epsilon_{1} }}$ is given by

10 $$ A_{{{\text{TA}},t, \epsilon_{1} , \epsilon_{1} }} = 1 + \left( {\frac{{\mathop \sum \nolimits_{{ \epsilon_{2} }}^{n - 1} L_{{ \epsilon_{2} , \epsilon_{1} ,t}} + \mathop \sum \nolimits_{{\epsilon_{2} }}^{n - 1} \dot{C}_{{ \epsilon_{2} , \epsilon_{1} ,t}} }}{{L_{{ \epsilon_{1} ,n,t}} }}} \right) \cdot \left( {1 - {\text{e}}^{{ - \frac{{L_{{ \epsilon_{1} ,n,t}} }}{{C_{{ \epsilon_{1} ,t}} }} \cdot \Delta {\text{t}}}} } \right). $$

To the non-diagonal entry $A_{{{\text{TA}}, \epsilon_{1} , \epsilon_{2} }}$, where $\epsilon_{1} \ne \epsilon_{2}$, the following applies:

11 $$ A_{{{\text{TA}},t, \epsilon_{1} , \epsilon_{2} }} = - \frac{{L_{{ \epsilon_{2} , \epsilon_{1} ,t}} + \dot{C}_{{ \epsilon_{2} , \epsilon_{1} ,t}} }}{{L_{{ \epsilon_{1} ,n,t}} }} \cdot \left( {1 - e^{{ - \frac{{L_{{ \epsilon_{1} ,n,t}} }}{{C_{{ \epsilon_{1} ,t}} }} \cdot {\Delta t}}} } \right). $$

The vector $b$ containing the temperature differences equals to

12 $$ b_{{{\text{TA}},t}} = \left( {\begin{array}{*{20}c} {T_{1,t} \cdot {\text{e}}^{{ - \frac{{L_{1,n,t} }}{{C_{1,t} }} \cdot {\Delta t}}} + \frac{{P_{V,1,t} }}{{L_{1,n,t} }} \cdot \left( {1 - {\text{e}}^{{ - \frac{{L_{1,n,t} }}{{C_{1,t} }} \cdot {\Delta t}}} } \right)} \\ \vdots \\ {T_{{n_{ \epsilon } - 1,t}} \cdot {\text{e}}^{{ - \frac{{L_{n - 1,n,t} }}{{C_{n - 1,t} }} \cdot {\Delta }t}} + \frac{{P_{V,n - 1,t} }}{{L_{n - 1,n,t} }} \cdot \left( {1 - {\text{e}}^{{ - \frac{{L_{n - 1,n,t} }}{{C_{n - 1,t} }} \cdot {\Delta t}}} } \right)} \\ \end{array} } \right). $$

In Eq. (12), the conductivity value $L_{ \epsilon ,n}$ is the denominator. For the node $\epsilon$, which is not connected to the ambient, the conductivity value $L_{ \epsilon ,n}$ equals $0 {\text{W}}/{\text{K}}$. To maintain mathematically stable results, these conductivity values are set to $10^{ - 9} {\text{W}}/{\text{K}}$ instead.

For the transient calculation, the rotational speeds and torques of the input shaft are given by a set load case. These variables of the remaining components and the power losses are calculated in the gearbox design tool in advance, making them accessible for all time steps.

### Analysis of oil distribution using smoothed particle hydrodynamics

Considering the oil–air mixture within the gearbox is of essential importance for the quality of the model, as this medium is responsible for a significant part of the heat transport. Its complex flow characteristic depends on the current operating point as well as the specific geometric gearbox configuration. A simulative approach using SPH is presented to constitute a uniform method for the oil distribution calculations of splash lubricated spur gear stages. Based on typical gearbox configurations, the influences of individual gearbox parameters on the oil distribution are examined and evaluated. In particular, sets of fluid simulations are performed to determine the respective effect of each parameter. The primary goal is to develop a concept for using oil simulation data in thermal network analysis. The basis is an expandable data structure in the form of look-up tables.

**Fig. 10:**
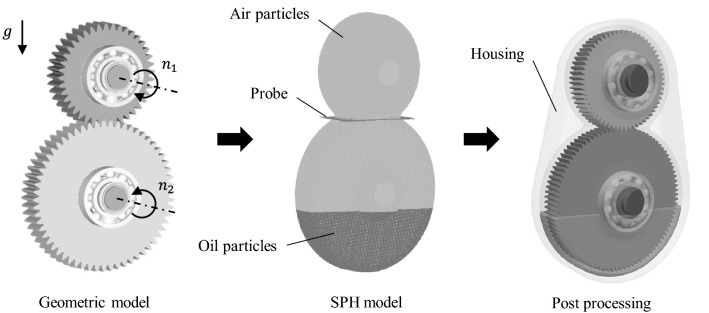
Modeling process for the SPH simulation

The gearbox model used for the SPH simulation always consists of a one-stage helical gearpair. The suitable housing is created with the presented geometric function. The gears are, respectively, positioned on the shafts. Four roller bearings enable rotation in the gearbox housing. Two radial shaft seals prevent the leakage of the lubricant. The position of the individual components within the gearbox system is shown in Fig. 10, which shows the basic modelling process for the fluid simulation.

The process is divided into three parts. The geometric model includes the generation and assembly of the gearbox components. Additionally, the boundary conditions are defined. The forces and speeds in the system domain are limited to the fundamental gearbox processes. In the case of the rotational speeds, only the involved gears and shafts are enabled. For the sake of simplicity, bearings and seals are assumed to be motionless. The gravitational acceleration $g$ of 9.81 m/s^2^ determines the overall domain force for the fluid system. The gravity also references the location of the oil sump inside the gearbox. The SPH model is derived from the geometric model. Table 3 depicts the basic simulation parameters.

**Table 3:** Simulation parameter used for SPH

Simulation parameter	Symbol	Value	Unit
Particle spacing	$\mathrm{d}x$	0.7	$\mathrm{mm}$
Simulation time	$t$	0.4–2.5	s
Particle output frequency	$\Delta t$	$\Delta t\le$ 1/100	s

For the generation of the particles, a universal particle spacing of 0.7 mm is assumed. The specific value is determined in preliminary experimental analysis by lubricant outlet tests and validated by literature [11]. In this case, the lubricant movements show appropriate fluid mechanics and ensure sufficient computing time. This leads to an approximate number of 5.7 million particles for the presented model. The simulation time for analysing the oil distribution depends on the respective configuration and operating point. To guarantee a suitable measurement resolution, the particle output frequency should be at least one hundredth of the chosen simulation time. The SPH modelling mainly includes the generation of the fluid particles. The initial positioning of the oil and air particles depends on their material properties and the predefined oil level. The densities and viscosities of the two fluids used in the simulations are defined in Table 4. Because of their temperature dependency, a typical operating temperature of 50 °C is assumed to specify these characteristics.

**Table 4:** Material properties of the gearbox components

Component	Material property	Value	Unit
Transmission component	Rigid body	–	–
Transmission oil (GL4) SAE 80 W	$\rho \left(T=50^\circ \mathrm{C}\right)$ $\eta \left(T=50^\circ \mathrm{C}\right)$	857.95 0.05019	$\mathrm{kg}/\mathrm{m}^{3}$ $\mathrm{Pa s}$
Air	$\rho \left(T=50^\circ \mathrm{C}\right)$ $\eta \left(T=50^\circ \mathrm{C}\right)$	1.09 1.967 × 10^–5^	$\mathrm{kg}/\mathrm{m}^{3}$ $\mathrm{Pa s}$

The solid components exhibit a larger density compared to the fluids. Since the focus is on investigating the oil distribution inside the gearbox, it is appropriate to simplify the model here. To reduce the complexity of the simulation model, all gearbox components are consequently defined as rigid bodies. In addition to the particle generation, the probe settings are specified. Probes serve as measurement tools to record the fluid flow during the simulation process. For the solving process, the software nanoFluidX is used. Based on a weekly compressible SPH formulation, it is created and optimised for use on clusters of GPUs.

For this reason, the simulations are running on the High Performance Computing (HPC) cluster at RWTH Aachen University. The computing node used consists of a Supermicro 1029GP-TR Superserver. Its specifications include a 2.1 GHz Intel Xenon Platinum 8160 processor and two NVIDIA Tesla V100 graphic units with 16 GB VRAM. The applied Riemann solver is suitable for multiphase flow problems regarding aeration and windage effects. Parallelised algorithms utilise the Lagrangian particle method. Post-processing enables a graphical illustration of the fluid simulation. Figure 10 (right) also shows a rendered image of an initial gearbox state.

**Fig. 11:**
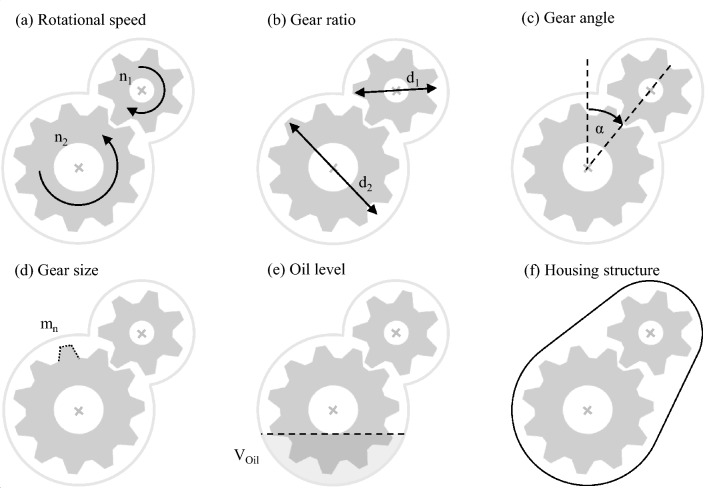
Variation parameters of the oil flow simulation using the SPH method

The accomplished investigations of the fluid system depending on the respective gearbox configuration are presented below. To ensure the comparability between the several fluid simulations, only one gearbox parameter is varied at a time. The basis of the models is general defined stationary parameters, which have constant values for all gearbox configurations. These include a constant axle distance of 150 mm for the gearpairs, a gear width of 30 mm, equal designs for all bearings and seals and a constant housing structure. Several characteristic gearbox parameters are considered to analyse an extensive range of influencing factors for oil distribution. Figure 11 illustrates these examined parameters.

The gears' rotational speeds (a) depend on the prevailing load case and initiate the lubricant flow inside the gearbox. To cover a variety of load cases, speed series are simulated for all considered parameters. The gear ratio (b) investigates the effect of the gear size on the lubricant system. Due to the gear positions (c), different angles of the gear arrangements are studied. This parameter mainly affects the location of the oil sump. The module characterises the gear tooth size (d). Additional investigations of the influence of the oil level (e) inside the gearbox and different housing structures (f) are simulated prospectively. The individual parameter values are listed in Table 5.

**Table 5:** Range of examination of the gearbox parameters within the oil flow simulation

Test series	Symbol	Value	Unit
(a) Rotational speed	$n$	0–8000	rpm
(b) Gear ratio	$i$	1, 1.5, 2, 3	–
(c) Gear angle	$\alpha$	0, 45, 90, 135, 180, 225, 270, 315	°
(d) Module	${m}_{n}$	2, 3, 5	$\mathrm{mm}$
(e) Oil volume	$V$	0.1, 0.2, 0.4	${\mathrm{dm}}^{3}$

**Fig. 12:**
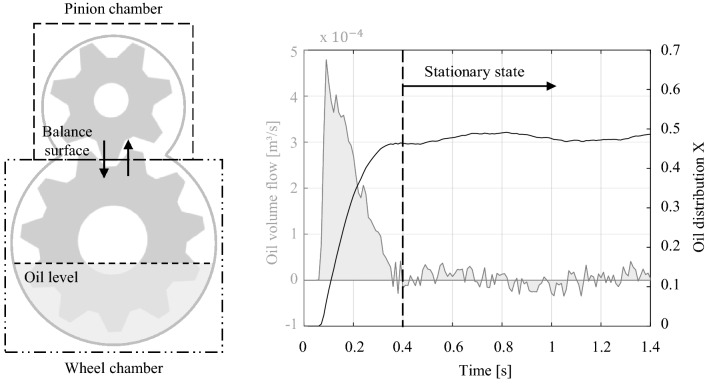
Evaluation procedure of the simulation results

The results of the fluid simulations provide the basis for the distribution calculations during the thermal analysis. Following, the applied measurement process of the oil flow is presented. To ensure a comparable calculation, all simulation models require a consistent evaluation approach. Therefore, the spur gear stage is divided into two balance areas, as shown in Fig. 12. One area includes the pinion gear, whereas the wheel characterises the other region.

Consequently, the analysis surface is located directly in the tooth contact of the gearpair. During the simulation process, the oil flow crossing the defined probe plane is being recorded. This sets up the basis for the determination of the distribution values. The volume of each balancing area depends on the gearbox configuration. In this regard, the parametrisation limits the maximum filling volumes of the individual oil chambers.

The measured oil flow shows significant characteristics for the constant rotational speeds of the gears. Figure 12 describes an exemplary plot of these measurements over the simulation time to clarify the correlation of the lubricant flow and its distribution. The oil distribution *X* indicates the ratio of the oil volume in the pinion chamber to the total oil volume in the gearbox. The flow characteristic can be divided into two phases. At the beginning of the simulation, a large oil volume flow over the balancing surface is recognisable, describing the start-up phase (0–0.4 s). The lubricant located in the oil sump is set in motion by the movement of the gears. This leads to initial oil distribution in the two defined oil chambers. As the simulation progresses, the oil flow characteristic shifts to oscillate around the zero line (0.4–1.4 s). This phase describes a stationary state in which the oil distribution in the gear stage remains approximately the same. The stationary state of each simulation defines the resulting distribution value of the applied gearbox parameter configuration. Therefore, a specific distribution value can be generated that describes a gearbox parameter's influencing factor depending on the rotational speed. Table 6 demonstrates the simulated oil distribution results for an exemplary speed series. The considered gearbox configuration has an oil volume of 0.2 dm^3^, a gear ratio of 2, a gear angle of 0°, a module of 2 mm and a default housing design. The viewed rotational speed range 0–8000 rpm of the pinion gear is divided into six separated support points. This gradation of simulated gearbox input speeds was deemed sufficiently detailed within an extensive simulation study. The aim of this study was to provide a sufficiently broad approach for the further parameter variations. This ensures that the necessary interpolations provide a good approximation for the subsequent analysis of deviating gearboxes.

**Table 6:**
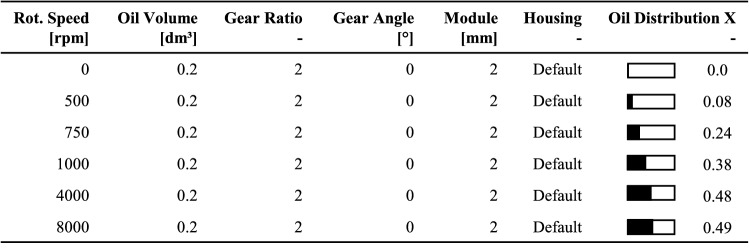
Evaluation of a simulation series under variation of the gearbox speed

**Fig. 13:**
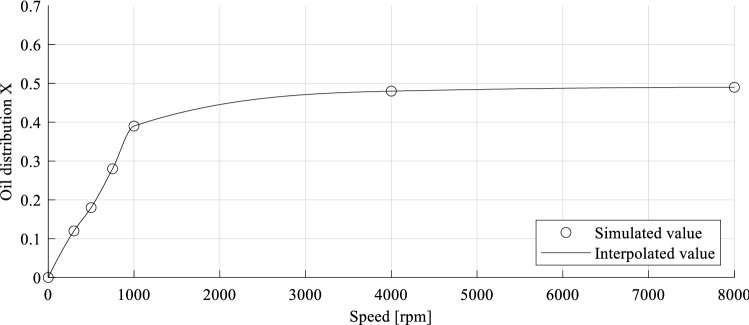
Interpolated oil distribution over total speed range

As a result of gravity, a lower system speed leads to a less efficient oil distribution *X* of the pinion chamber. Consequently, the amount of lubricant in this oil chamber decreases. A rotational speed of 500 rpm results in a distribution value of 8%. At higher speeds, the distribution approaches uniformity. For a rotational speed of 4000 rpm, the oil distribution *X* is 48%. Based on the discrete simulation results for the considered rotational speeds, complete distribution-speed curves are calculated by interpolation methods. To cover a range of load cases, intermediate values can be approximated. The interpolated oil distribution for the speed series data from Table 6 is plotted in Fig. 13 over the total speed range.

The characteristic of the speed-dependent oil distribution shows that a rapid increase is noticeable in the lower speed range (0–1000 rpm). Whereas for higher speeds (> 4000 rpm), a nearly constant distribution level proceeds. Between these two speeds, the oil distribution within the gearbox changes from an inhomogeneous to a roughly homogeneous distribution. By simulating speed series for several gearbox configurations, it is possible to generate a basis of distribution data, which allows for approximation of the fluid dynamics for any load case. The gearbox configuration is defined by its parameter settings. To give an overview of the influence of each examined gearbox parameter on the oil distribution, Table 7 compares the minimal parameter value to its maximum value. As a basis for the comparison, a default parameter set is predefined. It includes a rotational speed of 1000 rpm, an oil volume of 0.2 dm^3^, a gear ratio of 2, a gear angle of 0°, a module of 2 mm and a default housing layout. By changing only the respective parameter, the influence on the oil distribution *X* can be approximated.

**Table 7:**
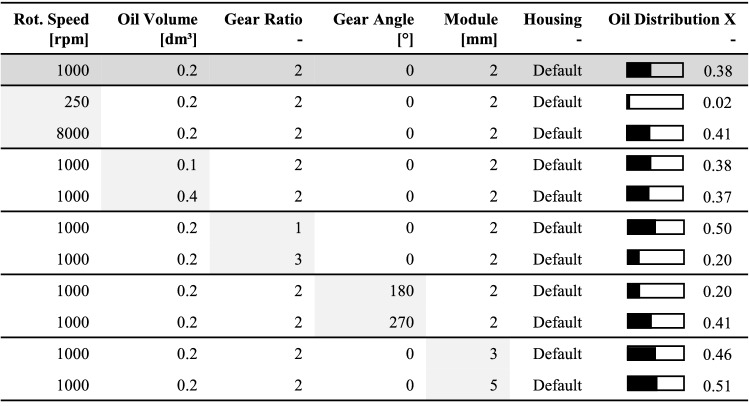
Extract of the results of all varied gearbox parameters within the oil flow simulation

**Fig. 14:**
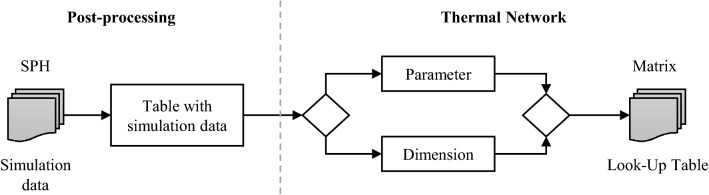
Process of integrating the oil flow simulation results into the thermal network calculation in MATLAB

The difference between the two oil distribution values for each parameter limit indicates the influence of its variation. Considering the default gearbox configuration, the rotational speed shows the highest impact on the lubricant distribution $\left( {\Delta X = 0.39} \right)$. The gear ratio and the mutual position of the spur gears have a significant effect on the distribution. $\left( {\Delta X \approx 0.21 - 0.30} \right)$. A minor influence is recognisable for varying the gear tooth size through the considered modules $\left( {\Delta X = 0.05} \right)$. The oil volume shows a negligible impact on the relative oil distribution $\left( {\Delta X = 0.01} \right)$. However, the total oil mass is crucial for the thermal analysis in the gearbox system. To take into account the oil distribution of the spur gear stage within the thermal network, access to the simulation data must be standardised and automated. The implementation process of the fluid simulation data into the thermal network process is shown in Fig. 14.

In this process of data preparation, a distinction is first made between the post-processing of the simulation data and the further processing of the simulation results within the thermal network. This subdivision helps to ensure that only the essential findings of the simulations are integrated into the thermal network. After evaluation of the simulation results, the steady-state values of the oil distribution are transferred to look-up tables, which allow interpolation during the calculation. This eliminates the need for the time-consuming integration of the simulation results into the thermal network.

## Application of the methodology

The application of the presented approach using the described method for estimating the oil distribution is carried out in the following chapter using the gearbox of the VW ID 3 as an example. Here, the system's thermal behaviour at a stationary load point and a dynamic load profile was examined. Since the selected test object is a series production gearbox, the analysis of internal system temperatures was not possible without extensive modifications. The analysis was limited to measuring selected housing temperatures.

### Validation of the method on the example of the VW ID 3

**Fig. 15:**
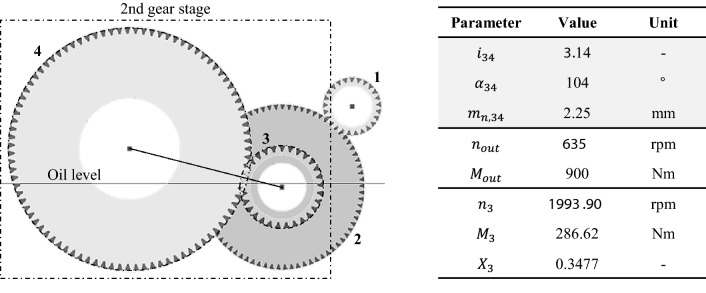
Simplified gearbox configuration of VW ID 3, parameterisation of 2nd gear stage

The information on the VW ID 3 gearbox necessary for the thermal network evaluation was obtained from public sources [6]. Since some information, such as the module, is not freely available, it was supplemented with values typical for the system application. The selected values can be seen in Fig. 15.

This single-speed gearbox consists of two gear stages. According to the configuration, the gears are positioned on three shafts. Gear 1 is connected to the input shaft, gears 2 and 3 are mounted on the centre shaft. The output shaft includes gear 4. For the following applications, the second gear stage is considered as an example.

The gear ratio defines the parameterisation of the second gear stage $i_{34}$, the gear stage angle $\alpha_{34}$ and the module $m_{n,34}$. The illustrated oil level describes the initial expression of the oil sump in the splash lubricated gearbox system. The used oil volume is 0.5 dm^3^. For the thermal network calculations, a stationary load case is examined. The constant speed defines the gearbox output $n_{{{\text{out}}}}$ and the load torque $M_{{{\text{out}}}}$. Consequently, the pinion gear 3 has the speed $n_{3}$ and torque $M_{3}$. The imposed load point results in a calculated oil distribution $X_{3}$ of 0.3477 using the featured SPH approach. This implies a lubricant distribution ratio for the second gear stage of approximately 35% for the pinion chamber and 65% for the wheel chamber.

**Fig. 16:**
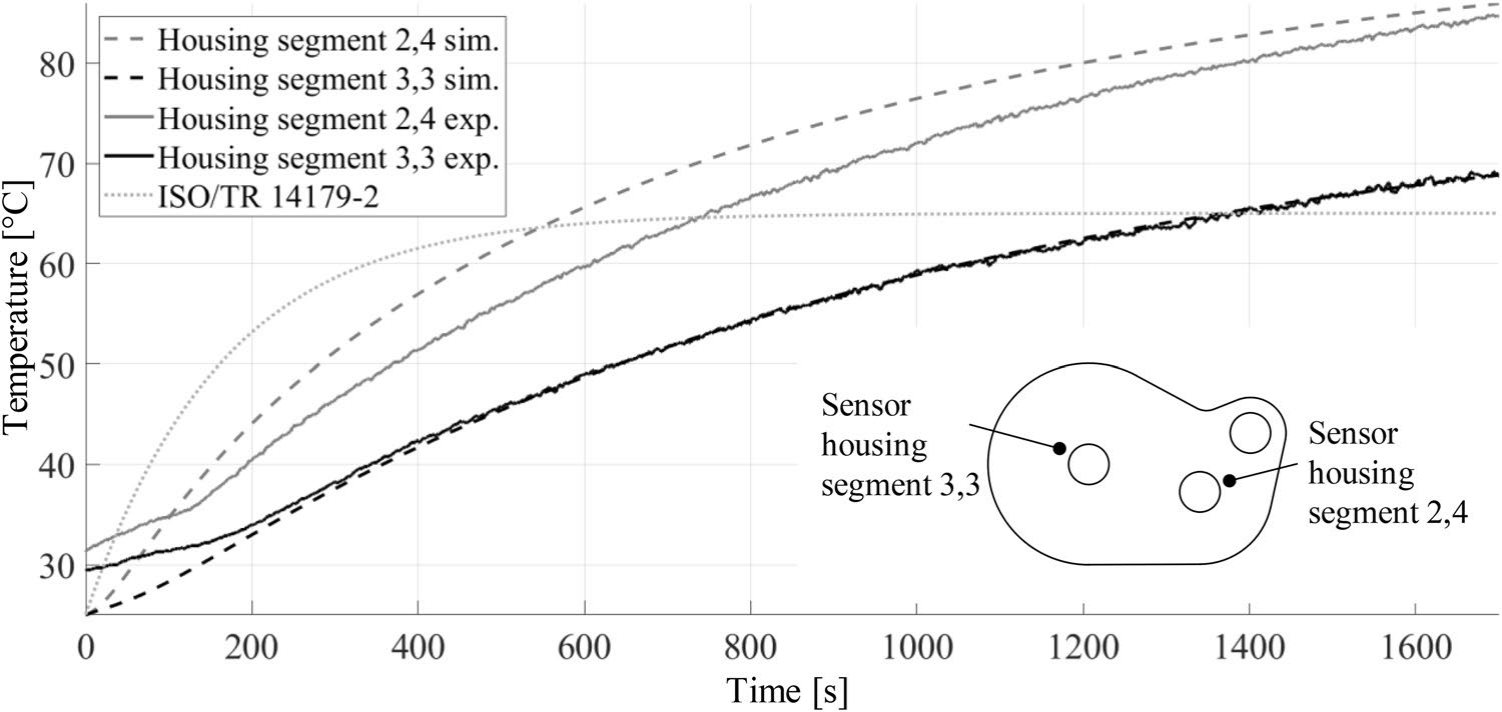
Simulated and measured temperature curves of the housing segments 2,4 and 3,3 for a stationary load case

The test of the gearbox of the VW ID 3 was conducted on a test bench at ika with two temperature sensors placed on the housing. The first was put on the centre shaft and the second on the output shaft, as shown in the bottom right corner in Fig. 16. Both were situated on the housing directly underneath a respective bearing. The positions of the temperature sensors correspond to the housing element, which are named with the indices 2,4 and 3,3 in the following. These result from the respective group and the element type. A detailed introduction of the index classification will be omitted due to its minor importance for the approach.

Figure 16 shows the simulated and the measured temperature curves for the heat up cycle with the given stationary load case. The test was performed at an ambient temperature of 24 °C. The difference between the two measured curves and the progression in the first 150 s are caused by multiple tests performed and the gearbox not being cooled down perfectly after each test. In this time range, the measured temperatures are approaching the simulated one asymptotically as expected, because the progression of the measured data is a superposition of a cool-down and a heat-up caused by the power losses. The automatically generated housing is used in the thermal analysis, and it does not represent the exact housing of the genuine gearbox. This is responsible for some of the deviations between measured and simulated values. Other reasons for the inequalities of the curves are inaccuracies in the calculations of the conductivity values, for example, in the Nusselt correlations.

The calculated oil temperature based on the standard ISO/TR 14179-2 is also shown in Fig. 16. Only the temperature for a stationary load case is calculated iteratively, so the heat up part is incomparable with the other parts. The end temperature of the standard calculation is lower than both simulated and measured end temperatures. This trend is underlined by the example given in ISO/TR 14179-2, for which the oil temperature of 59.7 °C was calculated, while the measured oil temperature was 70 °C. Considering the reasonable deviations, the model results show good accordance with the measured temperature data and the calculation according to the standard.

### Evaluation of the influence of a dynamic driving cycle on the heat development of a gearbox using the WLTP

**Fig. 17:**
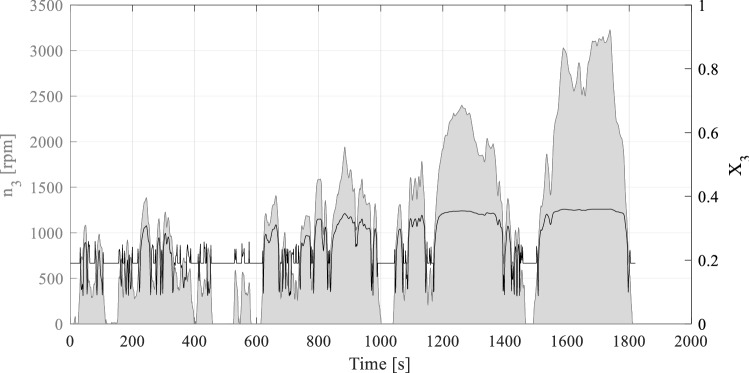
Calculation of the oil distribution $X_{3}$ over WLTP cycle within the thermal network analysis

The transient load case is characterised by load conditions changing over time. To consider a comparable load cycle, the standardised WLTC (Worldwide Harmonized Light-Duty Vehicles Test Cycle) is used as a reference. For each time step during the thermal network calculations, the lubricant distribution inside the gearbox has to be determined. The load-dependent oil distribution values are calculated for the defined oil spaces based on the developed SPH concept. Following the process is demonstrated using the example of the second gear stage of the VW ID 3. Therefore, Fig. 17 illustrates the oil distribution $X_{3}$ over the simulated WLTC. The rotational speed $n_{3}$ describes the load case for the associated pinion gear.

**Fig. 18:**
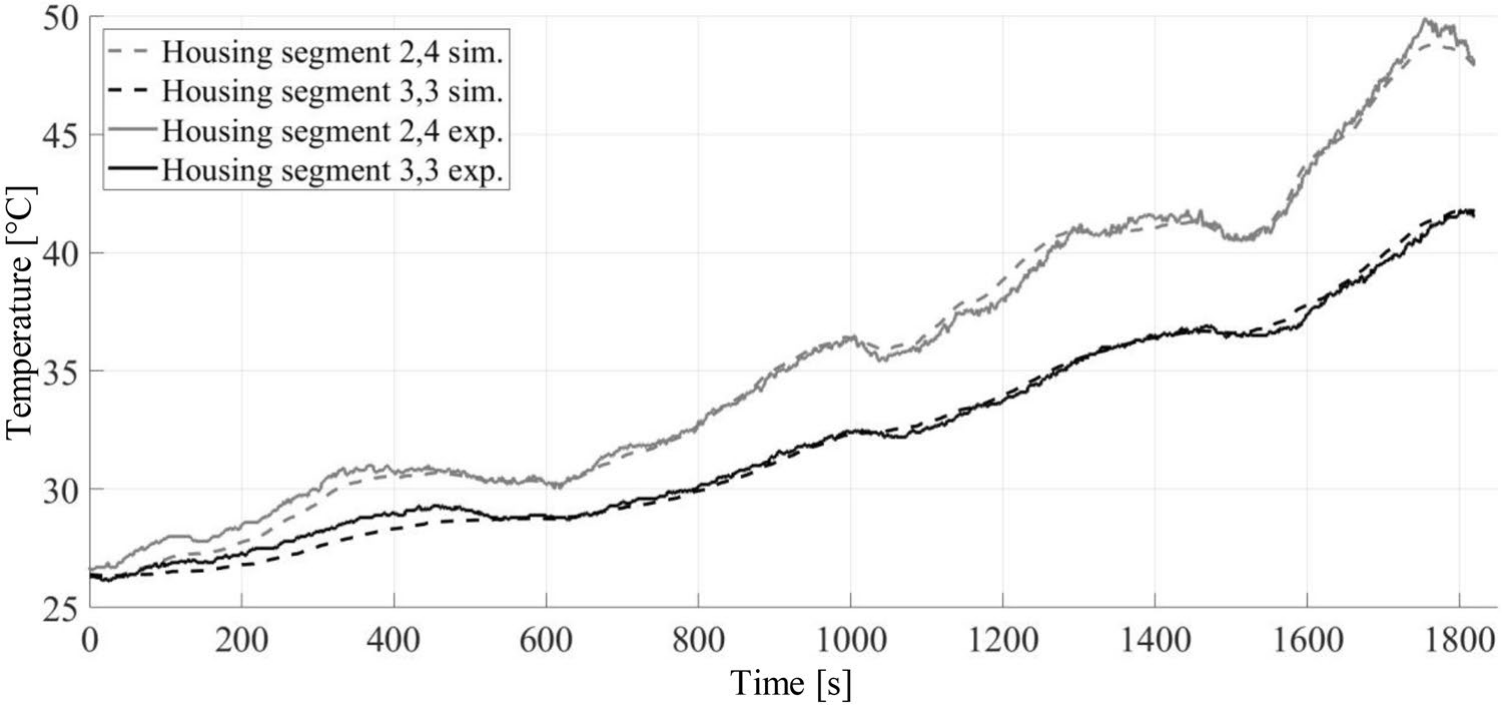
Simulated and measured temperature curves of the housing segments 2,4 and 3,3 for the given transient load case

The gearbox was tested on the test rig also for the transient load case and the two temperature sensors from the stationary temperature test were again used. The simulated and measured temperature curves are presented in Fig. 18.

For the measured data, the same problem as in the stationary load testing occurred, as the parts of the gearbox were not cooled down to ambient temperature. For better comparability, the start temperatures of all nodes in the model were set to 26.4 °C, and ambient temperature was set to 24 °C. The most significant deviation is notable in the first 400 s of the cycle with low temperatures. This is most likely caused by the start temperatures of the gearbox components described earlier. The temperature values for housing segment 2,4 are always higher than those of housing segment 3,3, since the components of group 3 rotate with lower speeds than components of group 2 in all cases, thus leading to less waste heat. The presented comparison demonstrates that the thermal analysis model results are in good compliance for transient load cases and stationary load cases and, therefore, can be used to identify critical thermal conditions in a gearbox.

## Discussion

The presented method enables an initial thermal system analysis to be carried out based on the results of an efficiency calculation and the associated gearbox configuration. This was applied to the VW ID 3 for a stationary and a dynamic load case and showed good correspondence with the measurement results. Estimating the oil distribution as a function of the load point used here led to a further refinement of the simulation results compared to a homogeneous distribution, especially for lower speed ranges. Based on these results, the methodology can be considered validated. A more detailed examination of the internal gearbox temperatures was not possible in the test setup carried out but is planned for subsequent test series to validate the internal system temperatures as well.

Using the method, an initial thermal assessment of the system can be carried out at an early stage of development. The time-step-based calculation of the component temperatures can also process transient load points. Since all the required thermal system variables are determined automatically using standardised gear elements, no detailed system design is necessary. Therefore, the analysis can already be conducted for the first design of a gearbox based on the characteristic gearbox parameters. This enables the developer to counteract possible hotspots during the subsequent design phase by introducing specific countermeasures, such as an oil injection point. The method also provides possibilities of implementing active lubrication. However, this approach is not explained here due to the validation system under consideration not featuring active lubrication. Nevertheless, this method offers an effective way to counteract local thermal hotspots.

Likewise, the data basis of the flow simulations makes it possible to consider the fundamental effects of different gearbox configurations and system speeds on the lubricant distribution within the gearbox stage without having to carry out an extensive system analysis as a comprehensive CFD of the gearbox. It should be emphasised here that the automated approach of housing generation makes this estimation possible in an early development phase. A CFD, on the other hand, requires a very detailed design of the gearbox. This relieves the developers of creating several housing designs, whereby it must be accepted that these housing designs may deviate from the later stiffened housing design, which may be suitable for casting.

Compared to the approaches presented in Sect. 2, the developed calculation method extends the approach of the thermal network for an automotive gearbox application. Compared to the previous analysis of test and industrial gearboxes, this extension enables the analysis of typical automotive housing designs. The significantly lower oil quantity and its substantially higher contact surface to the housing are decisive for the simulation result. In this application, the oil distribution within the gearbox is of particular importance for its heat balance. The necessary consideration of the distribution within the gearbox was integrated by incorporating the results of a comprehensive SPH simulation study in the thermal network approach. This is seen as a further distinction from the current state of the art.

At present, the method has been validated using a gearbox of an electric vehicle. This field of application is particularly suitable for the developed method, as higher system speeds also over longer time periods in comparison to combustion engine-propelled powertrains have to be considered for these systems. This leads to higher stresses, which influence the selection process of the gearbox components. With the current increasing electrification of commercial vehicles, a further field of application is added, as higher torques in addition to longer usage phases are required by these vehicles. To prevent damage to individual components, it is, therefore, advantageous for system developers to obtain knowledge about thermal hotspots at an early stage. Since a cooling system for temperature control of the electric machine is integrated into these electric drive systems anyway, adapting to an additional demand is possible with early planning.

## Conclusions and outlook

The presented method offers the possibility to perform an estimation of the heat balance based on a first gearbox configuration. In doing so, time-consuming design and simulation approaches are avoided through extensive data sets and simplified estimation approaches. Compared to the current approach contained in ISO/TR 14179-2, the developer can identify thermal hotspots within the system at a very early stage of development. The additional effort required for this is low, since thermal elements' elaboration and interconnections can be based on an automated equation generation. Compared to the gearbox detailing already needed for the efficiency calculation, only an estimation of the system housing and the oil distribution is necessary.

Since the thermal network generation is automated, it does not need user inputs once the gearbox configuration data is given. The thermal analysis can be combined with the gearbox topology design module of the ika design methodology. The topology is optimised with a genetic algorithm based on set weight factors for different characteristics [12]. The combination allows optimising a gearbox topology for the best possible thermal performance. This optimal available solution helps the developer find a compromise between thermal performance and other conflicting design factors, such as installation space, secondary consumers, and efficiency. This is particularly important for the compact package of an electrified driveline. These have much higher system speeds on the one hand, and on the other hand, place additional heat sources, such as the inverter and electric motor, near the gearbox.

The quality of the approach has already been demonstrated in the test using the VW ID 3 gearbox. The minor deviation of the simulated and measured housing temperatures allows the overall balance of the method to be considered successful. In additional experiments of the Concept ELV^2^ research project, the determined component temperatures were validated in addition to the housing temperatures presented in this paper. The addition of a simplified model of the electric motor and the inverter to the presented approach is currently state of the art. Comprehensive thermal modelling approaches already exist for these components, so that only their linkage with the approach developed here would have to be modelled for this extension. However, due to the extensive modelling effort of the presented method, this was not yet considered.
